# PARTICIPATION IN AN INTERGENERATIONAL SERVICE-LEARNING COURSE AND IMPLICIT BIASES

**DOI:** 10.1093/geroni/igz038.728

**Published:** 2019-11-08

**Authors:** Lori Kogan, Lori Kogan, Regina Schoenfeld-Tacher, James Oxley

**Affiliations:** 1 Colorado State University, Fort Collins, Colorado, United States; 2 North Carolina State University, Raleigh, North Carolina, United States; 3 NA, measham, England, United Kingdom

## Abstract

Biases against older adults and people with disabilities can lead to discriminatory behaviors. One way to better understand attitudes towards these populations is through the examination of implicit (unconscious) factors. This paper utilizes The Implicit Association Test, a computer-based categorization task designed to assess implicit or unconscious attitudes, to assess the impact of an intergenerational service-learning course created to support the human animal bond between vulnerable pet owners and their companion animals. This study, using the Wilcoxon signed-rank test, assessed the impact of college students’ interactions with older pet owners on these students’ implicit attitudes. Pre- and post-assessment of participating students found statistically significant decreased biases towards older people and people with disabilities after completing the course (p=.032). Results from this study suggest that participating in an intergenerational service-learning course centered around the human animal bond can positively affect implicit attitudes towards older adults or people with disabilities.

